# A machine learning model for grade 4 lymphopenia prediction during pelvic radiotherapy in patients with cervical cancer

**DOI:** 10.3389/fonc.2022.905222

**Published:** 2022-09-15

**Authors:** Zhiyuan Xu, Li Yang, Hao Yu, Linlang Guo

**Affiliations:** ^1^ Clinical Oncology Center, The University of Hong Kong - Shenzhen Hospital, Shenzhen, China; ^2^ Shenzhen Key Laboratory of Translational Research on Recurrent/Metastatic Cancer, The University of Hong Kong - Shenzhen Hospital, Shenzhen, China; ^3^ Shenzhen Institute of Advanced Technology, Chinese Academy of Sciences, Shenzhen, China; ^4^ Department of Pathology, Zhujiang Hospital, Southern Medical University, Guangzhou, China

**Keywords:** cervical cancer, machine learning model, lymphopenia, prediction, pelvic radiotherapy

## Abstract

**Background/purpose:**

Severe lymphopenia during pelvic radiotherapy (RT) predicts poor survival in patients with cervical cancer. However, the risk of severe lymphopenia has not been well predicted. We developed a machine learning model using clinical and dosimetric information to predict grade 4 (G4) lymphopenia during pelvic RT in patients with cervical cancer.

**Methods:**

This retrospective study included cervical cancer patients treated with definitive pelvic RT ± induction/concurrent chemotherapy. Clinical information and a set of dosimetric parameters of external beam radiotherapy plan were collected. G4 lymphopenia during RT, which was also referred to as G4 absolute lymphocyte count (ALC) nadir, was defined as ALC nadir <0.2 × 10^9^ cells/L during RT according to Common Terminology Criteria for Adverse Events (CTCAE) v4.03. Elastic-net logistic regression models were constructed for the prediction of G4 lymphopenia during pelvic RT using a repeated cross-validation methodology.

**Results:**

A total of 130 patients were eligible, and 43 (33.1%) patients had G4 lymphopenia during RT. On multivariable analysis, G4 ALC nadir was associated with poor overall survival (OS) [hazard ratio (HR), 3.91; 95% confidence interval (CI), 1.34–11.38, p = 0.01]. Seven significant factors [Eastern Cooperative Oncology Group (ECOG) performance score, pre-RT hemoglobin, pre-RT lymphocytes, concurrent chemotherapy, gross tumor volume of regional lymphadenopathy (GTV_N volume), body volume, and maximum dose of planning target volume receiving at least 55 Gy (PTV_5500 Dmax)] were obtained by elastic-net logistic regression models and were included in the final prediction model for G4 ALC nadir. The model’s predicting ability in test set was area under the curve (AUC) = 0.77 and accuracy = 0.76. A nomogram of the final predicting model was constructed.

**Conclusions:**

This study developed and validated a comprehensive model integrating clinical and dosimetric parameters by machine learning method, which performed well in predicting G4 lymphopenia during pelvic RT for cervical cancer and will facilitate physicians to identify patients at high risk of G4 lymphopenia who might benefit from modified treatment approaches.

## 1. Introduction

Cervical cancer is the fourth most frequently diagnosed cancer and the fourth leading cause of cancer death in women (1). Pelvic radiotherapy (RT) plays an integral part in the treatment of locally advanced cervical cancer (2), but it can also result in toxicities, including effects on host immunity. A higher radiation dose to immune cells was reported to be associated with poor treatment outcomes in patients with non-small cell lung cancer (NSCLC) (3). Lymphocytes, one of the most important components of the immune system, are especially critical in mediating cellular immunity against malignant tumor cells. In cervical cancer patients treated with concurrent chemoradiotherapy (CCRT), the incidence of grade 3 (G3) and grade 4 (G4) lymphopenia during CCRT, graded by Common Terminology Criteria for Adverse Events (CTCAE), reached as high as 73% and 16%, respectively, and G4 lymphopenia was associated with poor survival (4). Although it has clinical significance, the risk of G4 lymphopenia has not been well predicted in cervical cancer patients.

Many factors were reported to be associated with lymphopenia during RT. Radiation *per se* is among the most important risk factors for lymphopenia because lymphocytes continuously traverse the irradiated field and are extremely sensitive to radiation (5). The modeled RT dose to peripheral lymphocytes were associated with lymphopenia in patients treated with RT (6). Radiation field size, dose per fraction, and fraction number are all correlated with risk of lymphopenia (7). Dose–volume parameter (volume receiving at least 40 Gy) of the pelvic bone marrow was associated with a higher risk of acute G3 [odds ratio (OR)=1.018] or late grade 2 (G2) lymphopenia (OR=1.005) in prostate cancer patients treated with RT (8). The RT dose to the large blood vessels, bone, and whole body were also correlated with lymphopenia (6). Besides these dose–volume parameters, our previous study demonstrated that the International Federation of Gynecology and Obstetrics (FIGO) stage, pre-treatment lymphocyte, and pre-treatment hemoglobin were significantly associated with lymphopenia during CCRT in cervical cancer patients. Other studies showed that baseline lymphocyte had an important role in predicting lymphopenia during RT (8, 9). Integrating both dosimetric and clinical information might improve the prediction performance for lymphopenia.

Machine learning (ML), one of the most relevant subsets of artificial intelligence (AI) in medicine, mainly focuses on making as accurate predictions as possible. Compared with traditional statistical methods, ML could be more suited in highly innovative fields with a huge bulk of data (10). By using deep ML method to integrate dosimetric and clinical information, Cong Zhu et al. (9) developed a model to predict G4 RT-induced lymphopenia in patients with esophageal carcinoma with area under the curve (AUC) at 0.831, accuracy at 0.769, and precision at 0.670.

At present, ML method has not been widely applied in the prediction of G4 lymphopenia during pelvic RT for cervical cancer. By integrating both clinical factors and a set of dosimetric parameters, this study aimed to build an ML model to predict G4 lymphopenia during pelvic RT in patients with cervical cancer, with the hope to aid the physician’s decision-making process in clinical practice.

## 2. Materials and methods

### 2.1 Patients

This study was approved by the institutional ethics committee of the University of Hong Kong-Shenzhen Hospital [No (2022).020], and informed consent form from each patient for this study was waived. A cohort of patients diagnosed with cervical carcinoma from January 2015 to February 2021 in the University of Hong Kong-Shenzhen Hospital was selected for this study. Patients were included if they met the following criteria: 1) ≥18 years old; 2) newly diagnosed, pathology-confirmed cervical carcinoma; 3) FIGO stage (2018) IB-IVB (only stage IVB with oligo-metastases scheduled for radical pelvic RT were included); 4) major treatment was external beam radiotherapy (EBRT) followed by brachytherapy (BT) or stereotactic body radiotherapy (SBRT, if BT was contraindicated or declined) with or without induction or concurrent chemotherapy; and (5) complete blood counts (CBCs) were tested before and weekly during RT. Patients were excluded if they had the following: 1) cervical small cell carcinoma; 2) concomitant secondary primary malignant tumor; 3) acquired immune deficiency syndrome (AIDS); 4) pelvic RT in recurrent or adjuvant settings; and 5) did not complete planned EBRT.

### 2.2 Radiation therapy

All patients received pelvic EBRT followed by BT or SBRT. The simulation computed tomography (CT) scans for EBRT were taken with 3-mm slices from the interspace between thoracic vertebra 9 and 10 to the upper one-third of the femur. EBRT techniques were RapidArc or three-dimensional conformal radiotherapy (3D-CRT). For RapidArc, gross tumor volume (GTV) of the primary tumor (P) and regional pathological lymph nodes (N) detected by physical examination, simulation CT, pelvis magnetic resonance imaging (MRI), or positron emission tomography (PET)/CT were denoted as GTV_P and GTV_N, respectively. CTV_4500 [clinical target volume (CTV) receiving prescribed dose of ≥45Gy], including cervix, bilateral parametrium, uterus, part of vagina, and pelvic lymphatics; CTV_5500 (CTV receiving prescribed dose of ≥55 Gy), pelvic GTV_N + 3 mm margin; CTV_5750 (CTV receiving prescribed dose of ≥57.5 Gy), retroperitoneal GTV_N + 3 mm margin; PTV_4500, PTV_5500, and PTV_5750 [planning target volume (PTV) receiving prescribed doses of ≥45, ≥55, and ≥57.5 Gy], CTV_4500, CTV_5500, and CTV_5750 + 5 mm margin, respectively. Prescription dose was delivered as 45 Gy in 25 fractions (45 Gy/25 Fr) to PTV_4500 with a simultaneous integrated boost (SIB) of 55 Gy to PTV_5500 or 57.5 Gy to PTV_5750. For 3D-CRT, two sequential phases were adopted: 45 Gy/25 Fr to whole pelvis as phase I; boosting to pelvic wall with 16 Gy/8 Fr for FIGO IIIB or 10 Gy/5 Fr for other stages as phase II. All EBRT was delivered daily, five fractions per week. CT- or MRI-guided BT was started 3–4 weeks after the initiation of EBRT with ^192^Ir (iridium) high-dose rate, once a week for a total of 4 weeks. Cumulative equivalent doses in 2 Gy/Fr (EQD2) of > 84 Gy for stage IB–IIIA and >90 Gy for ≥ stage IIIB were set to cervical primary tumor.

### 2.3 Chemotherapy

Concurrent cisplatin (40 mg/m^2^) was given weekly during EBRT for up to 5–6 weeks. If creatinine clearance ≤50 ml/min, carboplatin at the dose of AUC = 2 mg/ml/min was given weekly as an alternative. Induction chemotherapy (IC) with paclitaxel and carboplatin was given if anticipated RT waiting time exceeded about 3 weeks. For patients aged over 70 or with FIGO stage IB1, no chemotherapy was recommended.

### 2.4 End points and dose–volume histogram metrics

Absolute lymphocyte count (ALC) was measured as ×10^9^ cells/L and graded by CTCAE v4.03. G4 lymphopenia during RT, which was also referred to as G4 ALC nadir, was defined as ALC nadir < 0.2×10^9^ cells/L during RT. Progression-free survival (PFS) was the time between the initiation of RT and the date of disease progression or death from any cause. Overall survival (OS) was the time between the initiation of RT and the date of death from any cause. Dose–volume histogram (DVH) metrics of both tumor targets and organs at risk (OARs) during EBRT were extracted directly from the Varian eclipse treatment planning system (version15.0, External Beam Planning, Varian) with anisotropic analytical algorithm. The tumor targets of interest included GTV_P, GTV_N, CTV_4500, PTV_4500, and PTV_5500. The OARs of interest included body (defined as the part of body within the range of simulation CT scan for EBRT) and bones (defined as bones within 2 cm beyond PTV). For each structure, the whole volume [in cubic centimeter (cc)], maximum dose (Dmax, in Gy), mean dose (Dmean, in Gy), and the percentage of the whole volume receiving ≥5, ≥10, ≥20, ≥30, ≥40, and ≥45 Gy (denoted as V5, V10, V20, V30, V40, and V45, respectively) were extracted.

### 2.5 Univariate and multivariable analysis

For the outcomes of OS and PFS, G4 ALC nadir and all clinical characteristics were analyzed by the univariate and multivariable Cox proportional hazards regression (cox-PH) models. Kaplan–Meier product-limit estimates with time-to-event curves were generated. To classify factors associated with G4 ALC nadir, all clinical characteristics and DVH metrics from tumor targets and OARs were analyzed between patient groups with or without G4 ALC nadir by the univariate logistic regression method.

### 2.6 Elastic-net logistic regression modeling

Elastic-net logistic regression is a type of penalized logistic regression (11, 12). Elastic-net uses both L1 and L2 norm penalty on the regression covariates and uses a mixing parameter that defines the proportion (alpha parameter) of penalty applied to the covariates between both L1 and L2 norms. Taken together, the elastic-net regression method allows retention of correlated covariates and also regularizes model predictors in a manner that allows for improved prediction performance. The risk factors selected from clinical characteristics and DVH metrics by elastic-net logistic regression models were applied to construct the multivariable logistic regression model.

Elastic-net logistic regression models were constructed for G4 ALC nadir prediction using a repeated cross-validation (CV) methodology to approximate the models’ generalization abilities when lacking an external validation dataset (13, 14). To determine the important features for G4 ALC nadir by elastic-net logistic regression models, we selected the best alpha parameter in one randomly separated train set as the first step; then, one elastic-net model was established in the train set and validated in the rest of the test set in 10-fold CV; finally, the 10-fold CV process was repeated 100 times in different held-out sets to estimate model mean efficacy [95% confidence interval (CI)], which is called repeated CV, considering to reduce overfitting in the small sample size. The statistically significant features were selected as the important features for G4 ALC nadir.

### 2.7 Statistical considerations

The Wilcoxon paired rank test was applied to compare the performances between two models. A p-value <0.05 was considered statistically significant in all statistical analysis. The Bonferroni correction was applied in multiple statistical testing. R software (version 4.0.2, R Development Core Team, Vienna, Austria) was used to conduct all statistical analyses. Elastic-net logistic regression modeling was implemented by the R package glmnet.

## 3. Results

### 3.1 Patient characteristics and clinical outcomes

Both pre-RT characteristics and clinical outcomes of the patients are listed in **Table 1**. A total of 130 patients formed the study cohort. The median age at diagnosis was 53 [interquartile range (IQR), 46–63] years. RapidArc was used in 79.2% of the patients. Twenty percent of patients had IC, and 83.8% received concurrent chemotherapy. The median (IQR) follow-up was 26.4 (14.2–41.6) months. The incidence of death, disease progression, local failure, regional lymph node metastasis, and distant metastasis during follow-up was 19.2%, 24.6%,11.5%, 3.8%, and 15.4%, respectively.

**Table 1:** Baseline characteristics and clinical outcomes of the patients.

Features	Categories	Median (IQR)or num (%)
Death	No	105 (80.8%)
	Yes	25 (19.2%)
Disease progression	No	98 (75.4%)
	Yes	32 (24.6%)
Post-RT local failure	No	115 (88.5%)
	Yes	15 (11.5%)
Post-RT regional LN metastasis	No	125 (96.2%)
	Yes	5 (3.8%)
Post-RT distant metastasis	No	110 (84.6%)
	Yes	20 (15.4%)
Age (years)		53 (46-63)
ECOG	0-1	114 (87.7%)
	2	16 (12.3%)
FIGO stage (2018)	I–II	36 (27.7%)
	III	84 (64.6%)
	IV	10 (7.7%)
Body mass index		23.1 (20.1-25.1)
RT technique	3D-CRT	27 (20.8%)
	RapidArc	103 (79.2%)
Induction chemotherapy	No	104 (80%)
	Yes	26 (20%)
Concurrent chemotherapy	No	21 (16.2%)
	Yes	109 (83.8%)
Pre-RT regional LN metastasis	No	45 (34.6%)
	Yes	85 (65.4%)
Pre-RT CBCs (×10^9^ cells/L)	Leukocytes	6.6 (5.1-8.1)
	Hemoglobin (g/L)	118 (103-130)
	Platelets	260 (216.5-316.8)
	Neutrophils	4.3 (3.1-5.6)
	Lymphocytes	1.7 (1.3-2.1)
	Monocytes	0.3 (0.2-0.4)

CBCs, complete blood counts; ECOG, Eastern Cooperative Oncology Group; FIGO, International Federation of Gynecology and Obstetrics; IQR, interquartile range; LN, lymph node; OS, overall survival; PFS, progression-free survival; RT, radiotherapy; 3D-CRT, three-dimensional conformal radiotherapy.

**Figure 1:**
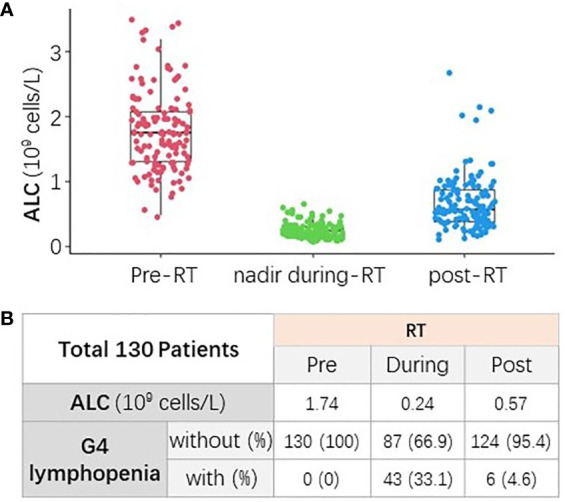
The change of absolute lymphocyte count (ALC) from baseline to post-RT. **(A)** ALC declined during RT and generally recovered to some extent at the completion of RT. **(B)** The median ALC and incidence of G4 lymphopenia at different time points (pre-, nadir during-, and post-RT).

ALC of all patients declined during RT and generally recovered to some extent at the completion of RT, as shown in **Figure 1A**. The median pre-RT ALC was 1.74×10^9^ cells/L. The counts declined during RT to the median ALC nadir as 0.24×10^9^ cells/L, and the median onset time of ALC nadir was 33 days from the initiation of RT. Finally, ALC partially recovered to the median counts of 0.57×10^9^ cells/L at the end of RT. The incidence of pre-, during-, and post-RT G4 lymphopenia were 0%, 33.1%, and 4.6%, respectively (**Figure 1B**).

### 3.2 G4 ALC nadir during RT was associated with poor clinical outcomes

**Figure 2:**
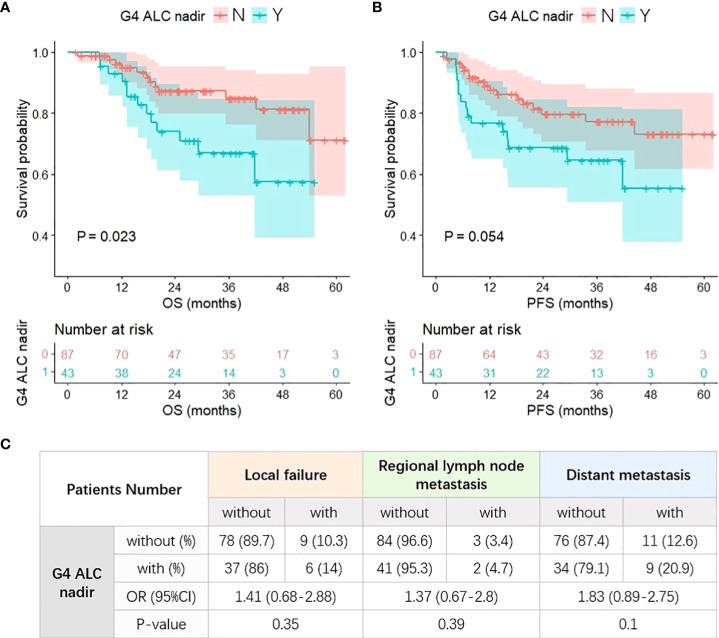
G4 ALC nadir and different treatment outcomes. **(A)** Patients with G4 ALC nadir had worse overall survival (OS) (p = 0.023). **(B)** Patients with G4 ALC nadir had worse progression-free survival (PFS) (p = 0.054). **(C)** The incidence and risk of local failure, regional lymph node metastasis, and distant metastasis after RT in patients with G4 ALC nadir.

During follow-up, there were a total of 25 deaths. G4 ALC nadir was seen in 33.1% of patients. Patients with G4 ALC nadir had worse OS (p = 0.023) and PFS (p = 0.054) than those without G4 ALC nadir as shown in **Figures 2A, B**. On univariate analysis, OS was significantly worse in patients with G4 ALC nadir than those without G4 ALC nadir [hazard ratio (HR), 2.44; 95% CI, 1.1–5.39; p = 0.03], and PFS was also worse in patients with G4 ALC nadir (HR, 1.96; 95% CI, 0.98–3.94; p = 0.05), as shown in **Table 2**. On multivariable analysis, patients with G4 ALC nadir still had significantly poor OS and a trend of poor PFS than those without G4 ALC nadir (HR, 3.91; 95% CI, 1.34–11.38; p = 0.01, and HR, 1.82; 95% CI, 0.75–4.42; p = 0.19, respectively). Although without statistical significance (p > 0.05), G4 ALC nadir showed a trend of promotion effects in the occurrence of local failure, regional lymph node metastasis, and distant metastasis after RT (OR, 1.41, 1.37, and 1.83, respectively) (**Figure 2C**).

**Table 2:** Univariate and multivariable analysis of potential factors associated with survivals.

Features	OS			PFS		
	HR	95% CI	p-value	HR	95% CI	p-value
Univariate analysis						
G4 ALC nadir: yes vs. no	2.44	1.1–5.39	0.03	1.96	0.98–3.94	0.05
Multivariable analysis						
G4 ALC nadir: yes vs. no	3.91	1.34–11.38	0.01	1.82	0.75–4.42	0.19
Age	0.01	2.09e−05–5.67	0.16	0.13	8.39e−04–18.91	0.42
ECOG: 2 vs. 0 and 1	1.81	0.46–7.17	0.4	1.75	0.49–6.25	0.39
FIGO stage III vs. I–II	0.8	0.22–2.91	0.74	1.26	0.43–3.67	0.68
FIGO stage IV vs. I–II	5.83	1.01–33.71	0.05	5.45	1.39–21.45	0.02
Body mass index	3.28e−03	4.37e−07–24.6	0.21	0.55	6.34e−04–478.03	0.86
RT technique: RapidArc vs. 3D-CRT	0.28	0.07–1.08	0.06	0.92	0.32–2.65	0.88
Induction chemotherapy: yes vs. no	1.4	0.35–5.67	0.64	0.77	0.24–2.45	0.65
Concurrent chemotherapy: yes vs. no	0.11	0.02–0.56	8.43e–03	0.79	0.21–2.98	0.73
Pre-RT regional LN metastasis: yes vs. no	3.78	0.88–16.3	0.07	1.11	0.38–3.3	0.85
Pre-RT leukocytes	6.92e−10	1.01e−27–4.72e+08	0.31	0.06	1.55e−14–2.27e+11	0.85
Pre-RT hemoglobin	9.30e−04	2.23e−07–3.87	0.1	0.04	7.04e−05–28.45	0.35
Pre-RT platelets	16.28	0.52–514.45	0.11	1.27	0.07–23.36	0.87
Pre-RT neutrophils	1.60e+09	1.5e−05–1.71e+23	0.2	60.81	1.41e−08–2.62e+11	0.72
Pre-RT lymphocytes	7.17e+04	0.04–1.33e+11	0.13	4.89	1.15e−04–2.08e+05	0.77
Pre-RT monocytes	1.18e−09	4.64e−18–0.3	0.04	1.13e−04	3.92e−10–32.55	0.16

ALC, absolute lymphocyte count; CI, confidence interval; ECOG, Eastern Cooperative Oncology Group; FIGO, International Federation of Gynecology and Obstetrics; G4, grade 4; HR, hazard ratio; LN, lymph node; OS, overall survival; PFS, progression-free survival; RT, radiotherapy; 3D-CRT, three-dimensional conformal radiotherapy.

### 3.3 Clinical and DVH characteristics and their correlations with G4 ALC nadir

The clinical characteristics were compared between the patient groups with or without G4 ALC nadir, and the univariate analysis results (ORs and p-values) are listed in **Supplementary Table S1**. Age, Eastern Cooperative Oncology Group (ECOG) performance status score, pre-RT hemoglobin, and pre-RT lymphocytes had protective effects from the occurrence of G4 ALC nadir (OR, 0.97, p = 0.03; OR, 0.11, p =0.04; OR, 0.97, p = 5.6e−3; OR, 0.22, p = 2.0e−4, respectively), while the usage of concurrent chemotherapy promoted the occurrence of G4 ALC nadir (OR, 5.73; p = 0.02). Body mass index (BMI) had protective (OR, 0.9; p = 0.06) and pre-RT regional lymph node metastasis had promotive (OR, 2.22; p = 0.06) effects from the occurrence of G4 ALC nadir with borderline significance.

All DVH metrics of interest were summarized in the format of median (IQR) in **Supplementary Table S2**. The radiation dosimetrics of different structures had high correlations (Pearson’s correlations > 0.5). There were little correlations among clinical characteristics and DVH dosimetrics, also among radiation dosimetrics and volumes, as shown in **Supplementary Figure S1**.

**Figure 3:**
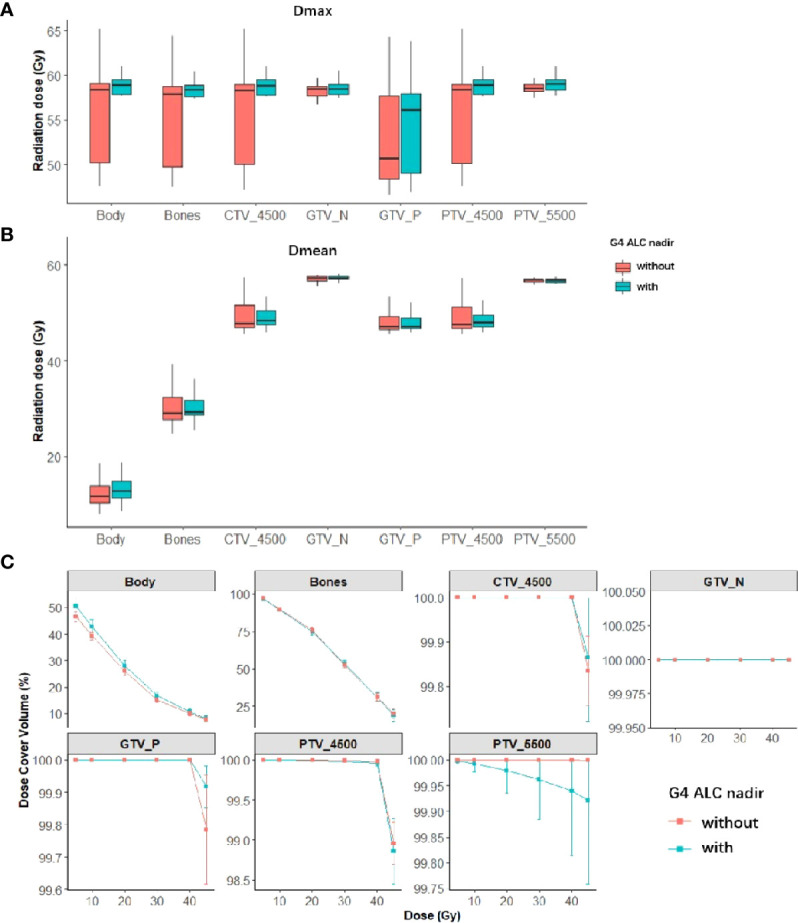
Summary of dose–volume histogram (DVH) metrics of both tumor targets and organs at risk (OARs) in patients with or without G4 ALC nadir (red is the summary of DVH metrics in patients without G4 ALC nadir; green is the summary of DVH metrics in patients with G4 ALC nadir). Panels **(A, B)** show the median (95%CI) of Dmax and Dmean of both tumor targets and OARs in patients with or without G4 ALC nadir. Panel **(C)** shows the relative volume (in percentage) covered by different dose levels of both tumor targets and OARs in patients with or without G4 ALC nadir.

The DVH dosimetrics of each structure were compared between the patient groups with or without G4 ALC nadir as shown in **Figure 3**, and univariate logistic regression analysis results of each DVH dosimetrics in each structure for G4 ALC nadir are listed in **Supplementary Table S3**. The volume of GTV_N was correlated with the occurrence of G4 ALC nadir (OR, 1.07; p = 0.01). The volume of GTV_P, all dosimetrics of PTV_5500, and the volume, V5, and V10 of the body showed a tendency to correlate with the occurrence of G4 ALC nadir (all p-values <0.1).

### 3.4 Elastic-net regression modeling for selecting risk factors affecting G4 ALC nadir

In searching grids from 0 to 1 step 0.05, the best alpha in the elastic-net logistic regression model with best performances was selected as 0.6 in one randomly separated train set. Then, elastic-net models were established in differently separated train sets and validated in the rest of the test sets in 10-fold CV for 100 iterations to summarize the prediction performances. As summarized in all models, mean AUC value in train sets was 0.84 (IQR, 0.82–0.86) and that in test sets was 0.76 (IQR, 0.69–0.83). The selected frequencies of all the 14 clinical characteristics and 63 DVH parameters in elastic-net regression models in 100 iterations bootstrapping are shown in **Supplementary Figure S2**.

**Figure 4:**
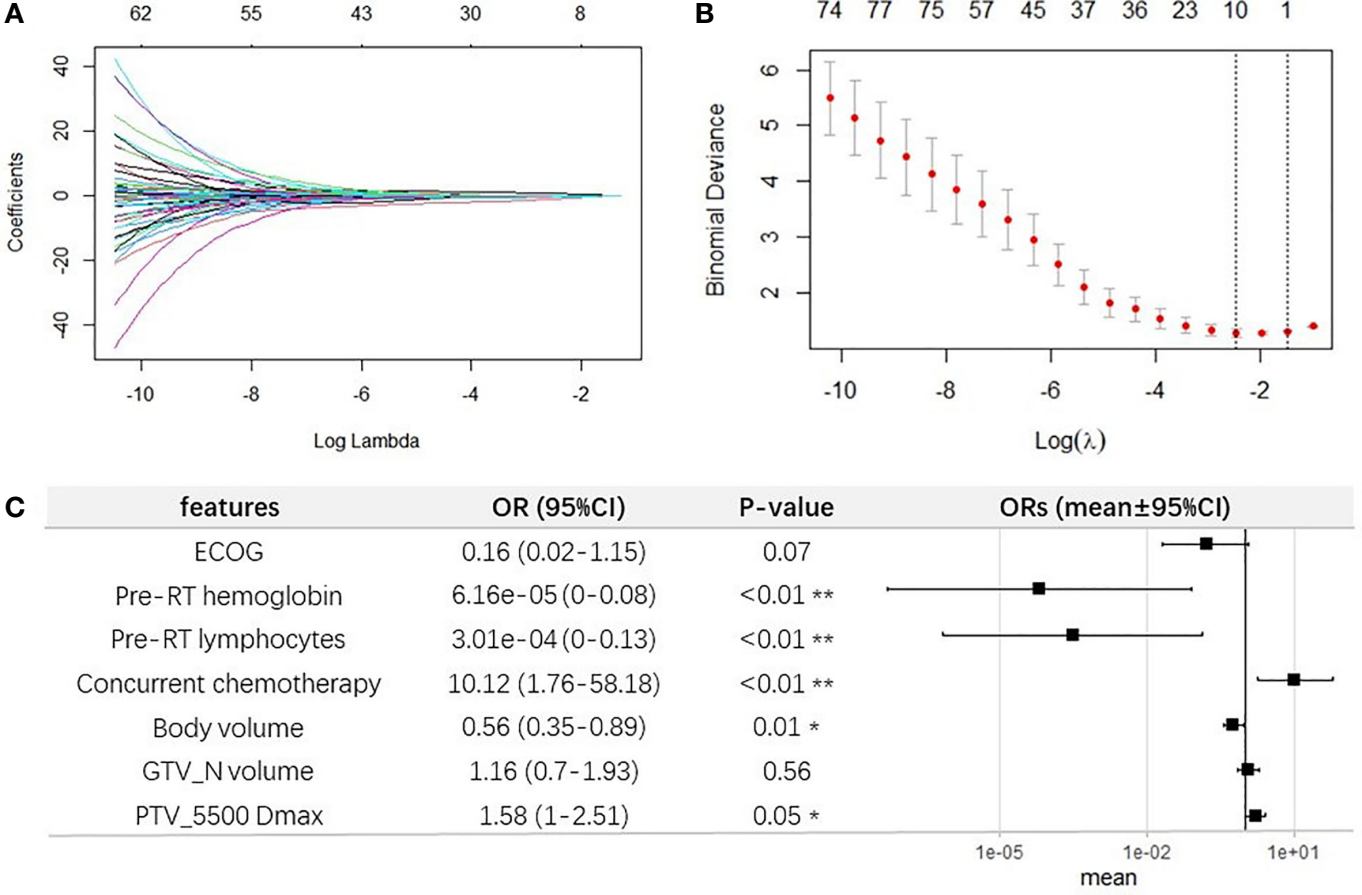
Elastic-net regression modeling for selecting important features for G4 ALC nadir. **(A)** Elastic-net coefficient profiles of all factors. **(B)** The selected optimal parameter (lambda) in the most moderate elastic-net model. **(C)** Forest plot of the seven selected important features for G4 ALC nadir.

The most moderate elastic-net model was selected as the final model. In building the model, the correlations between regression coefficients and lambda are shown in **Figures 4A, B**. The final model is shown in **Figure 4C**. Considering both the significance and selected frequency in bootstrapping, seven important risk factors were included in the final model (**Figure 4C**), including four clinical characteristics (ECOG, pre-RT hemoglobin, pre-RT lymphocytes, and concurrent chemotherapy) and three DVH parameters (GTV_N volume, PTV_5500 Dmax, and body volume).

### 3.5 Development and validation of the prediction model for G4 ALC nadir

The final multivariable logistic regression model was further constructed with these seven factors selected by elastic-net model in one seldomly separated train set, as shown in **Figure 4C**. Among clinical characteristics, ECOG (OR, 0.16; 95% CI, 0.02–1.15; p = 0.07), pre-RT lymphocytes (OR, 3.01e−04;95% CI,0–0.13; p < 0.01), and pre-RT hemoglobin (OR, 6.16e−05; 95% CI, 0–0.08; p < 0.01) were protective factors for occurrence of G4 ALC nadir, while concurrent chemotherapy was a promoting factor for G4 ALC nadir (OR, 10.12; 95% CI, 1.76–58.18; p < 0.01). Among DVH parameters, body volume played as a protective factor (OR, 0.56; 95% CI, 0.35–0.89, p = 0.01), while the GTV_N volume and PTV_5500 Dmax promoted the incidence of G4 ALC nadir (OR, 1.16; 95% CI, 0.7–1.93; p = 0.56; OR, 1.58; 95% CI, 1–2.51; p = 0.05, respectively). It was consistent with the coefficient in the final LASSO-net regression model.

**Figure 5:**
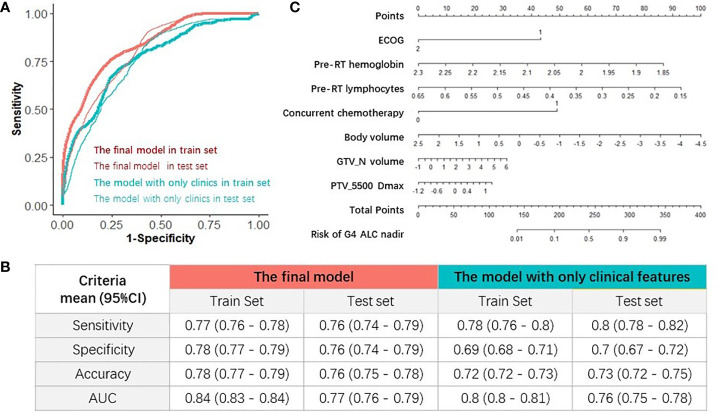
Evaluations of the models and nomogram for G4 ALC nadir prediction. **(A)** One example of receiver operating characteristic (ROC) curve of the prediction models in train and test sets. **(B)** Comparison of four evaluation criteria of different prediction models in train and test sets. **(C)** Nomogram of the final prediction model with seven parameters.

The final multivariable logistic regression model with seven important factors was compared with the multivariable logistic regression model with four clinical characteristics selected by elastic-net model. Their prediction abilities were testified in both train and test sets, as summarized in **Figure 5B**, and one example of AUC in the train and test sets was shown in **Figure 5A**. Four evaluation criteria, including sensitivity, specificity, accuracy, and AUC were all summarized and compared in **Figure 5B**. The final model with seven important factors had significantly higher AUC (mean, 0.84; 95% CI, 0.83–0.84) than the model with four clinical features (AUC mean, 0.8; 95% CI, 0.8–0.81) in train sets (a Wilcoxon paired rank test, p < 0.01), and the final model also had significantly higher AUC (mean, 0.77; 95% CI, 0.76–0.79) than the model with only four clinical features (mean, 0.76; 95% CI, 0.75–0.78) in test sets (a Wilcoxon paired rank test, p < 0.01). These results indicated that the DVH parameters improved the prediction performance for G4 ALC nadir. Finally, for the purpose of clinical usage in the future, the corresponding nomogram of the final multivariable logistic regression model with seven important factors for predicting G4 ALC nadir was plotted, as shown in **Figure 5C**.

## 4. Discussion

Lymphocytes are the most radiosensitive cells among the erythroid, myeloid, and lymphoid lineage with LD50 (lethal dose required to reduce the surviving fraction of lymphocytes by 50%) of only 2 Gy (5). RT-induced lymphopenia was common and correlated with poor survival in patients with different types of solid tumors, such as thoracic malignancies, brain tumors, head and neck cancers, and cervical cancer (15). In cervical cancer, the reported incidence of G4 lymphopenia during CCRT was 16%, and G4 lymphopenia could predict poor survival (4). The current study confirmed the results of previous studies. In our study, the incidence of G4 ALC nadir during pelvic (chemo)RT was as high as 33.1%, and G4 ALC nadir was associated with poor survival outcomes. Therefore, studies that focused on the prediction model for G4 lymphopenia are justified.

In this study, using the important risk factors selected from elastic-net models in machine learning framework, we developed and validated a multivariable logistic regression model for predicting G4 lymphopenia during pelvic (chemo)RT in cervical cancer patients with mean AUC = 0.84 (95% CI, 0.83–0.84), mean accuracy = 0.78 (95% CI, 0.77–0.79) in train sets and mean AUC = 0.77 (95% CI, 0.76–0.79), mean accuracy = 0.76 (95% CI, 0.75–0.78) in test sets. The final multivariable logistic regression model included four clinical characteristics (ECOG, pre-RT hemoglobin, pre-RT lymphocytes, and concurrent chemotherapy) and three DVH parameters (GTV_N volume, PTV_5500 Dmax, and body volume). Until now, we are not aware of any similar prediction models in cervical cancer. There are some other studies using machine learning algorithms to predict RT-induced lymphopenia in esophageal cancer (9, 16). Zhu and colleagues (9) constructed a novel deep learning model using dosimetric and clinical information to predict G4 lymphopenia during CCRT for esophageal cancer. Compared with their novel hybrid deep learning model in the train set, the AUC (0.84 vs. 0.831) and accuracy (0.78 vs. 0.769) of our final model were numerically similar (9). The performance of the final proposed model with both clinical and dosimetric factors was evaluated based on four prediction metrics and compared to the multivariable model with only clinical factors selected by elastic-net model. As expected, the model including both clinical and dosimetric factors outperformed the model including only clinical factors [mean AUC, 0.84 (95% CI, 0.83–0.84) vs. 0.8 (95% CI, 0.8–0.81), p < 0.01 in train sets, and mean AUC of 0.77 (95% CI, 0.76–0.79) vs. 0.76 (95% CI, 0.75–0.78), p < 0.01 in test sets]. With regard to potential clinical applications, we speculated that our model might play a role in the following clinical scenarios. First, as our model was totally based on pre-RT clinical and dosimetric parameters, it will enable physicians to assess EBRT plans for G4 lymphopenia risk and to identify patients at high risk who might benefit from modified treatment approaches and to guide modification of treatment approaches. Second, with the success of immunotherapy in solid tumors, the immunomodulatory effects of RT in conjunction with immune checkpoint blockade are currently under active investigation in cervical cancer (17). Lymphocytes are key effectors of immunotherapy, and lymphopenia was predictive for compromised efficacy of immunotherapy (18). It was reported that treatment-related severe lymphopenia was correlated with disease progression in NSCLC patients receiving consolidative immunotherapy after definitively chemoradiation (19). Applying lymphocyte-sparing RT has been recommended when combining RT with immunotherapy (20). RT-induced lymphopenia, which can be predicted using our model, should be one of the issues to take into consideration in designing clinical trials of RT combined with immunotherapy in cervical cancer (21).

In our proposed model, concurrent chemotherapy was a significant promote clinical factor for G4 ALC nadir (OR, 10.12; 95% CI, 1.76–58.18, p < 0.01), which was consistent with some previous studies (22, 23). In a study with large cohort of patients (N = 3,920) with different cancer types, the use of concurrent chemotherapy, particularly platinum compounds versus none, was associated with a lower ALC at end of RT (612 vs. 937 cells/ml, p < 0.001) (22). Another study with 711 patients who received definitive RT for NSCLC revealed that receipt of concurrent chemotherapy was associated with lower lymphocyte nadirs in multivariable analysis (p < 0.0001) (23). However, the contribution of concurrent chemotherapy to lymphocyte depletion is difficult to conclusively establish in studies demonstrating decreased ALC after CCRT. There were studies that showed that lymphopenia during CCRT was not significantly different among patients receiving different chemotherapy regimens, suggesting that no chemotherapy regimen *per se* was more likely to be cytotoxic to lymphocyte (24). In a study comparing effects of concurrent cisplatin administration during RT to RT alone on the immune function of patients with cervical cancer, administration of concurrent cisplatin might synergistically increase cytotoxic effects of radiation on tumor cells but did not alter the magnitude and the characteristics of radiation-induced immunosuppression (25). When patients received induction chemotherapy followed by consolidation chemoradiation, the drop in ALC occurred after consolidation therapy but not induction therapy, suggesting that induction chemotherapy did not play a major immediate role in causing lymphopenia (24, 26). Our study also showed that induction chemotherapy did not correlate with G4 ALC nadir during RT (OR, 1.09; p = 0.85). In esophageal cancer, neither induction chemotherapy nor the type of concurrent chemotherapy [e.g., taxane and 5-fluorouracil (5-FU) versus platinum and 5-FU or taxane and platinum or other] was associated with G4 lymphopenia (27). All these results suggest that the effect of concurrent chemotherapy on lymphopenia during RT is complex. More studies on the synergistic mechanism of chemotherapy and RT on the immune system are needed. Our study showed that ECOG was a protective factor from developing G4 ALC nadir. Patients with ECOG of 2 were less likely to develop G4 ALC nadir than those with ECOG of 0–1 (OR, 0.16; 95% CI, 0.02–1.15; p = 0.07). The possible reason for this result might be that patients with ECOG of 2 received less concurrent chemotherapy (p = 0.002).

Other two factors that promoted the incidence of G4 ALC nadir in our final model were GTV_N volume and PTV_5500 Dmax. In clinical practice, the dose level of 55 Gy was prescribed for metastatic locoregional lymph nodes. Both the two parameters indicated that the irradiation dose of the lymphatic system was positively correlated with G4 ALC nadir. Studies about the role of nodal irradiation for solid tumors on the reduction in circulating lymphocytes are scarce. Haas et al. studied the immunological effects of nodal irradiation for Hodgkin’s disease and showed that lymphoid irradiation (LI) was cytotoxic to peripheral blood T cells (28). They also postulated that the bone marrow outside the irradiation fields was a major source of T cells repopulating the peripheral blood after LI (28).

Among the clinical factors, pre-RT hemoglobin and lymphocytes were protective from developing G4 ALC nadir, which were consistent with results from previous studies (8, 9, 27, 29). Zhu et al. reported that patients with G4 lymphopenia during CCRT for esophageal cancer had lower level of baseline hemoglobin (12.94 vs. 13.28 g/dl, p = 0.008) and baseline ALC (1.42 vs. 1.78 x 10^9^/L, p <0.001) (9). Sini et al. also suggested that baseline ALC played an extremely important role in the development of lymphopenia in patients treated with pelvis RT for prostate cancer (8). Baseline ALCs below 1.83 x 10^9^/L were predictive of an enhanced probability of acute G3 lymphopenia (8). Recent studies found that erythroid cells can regulate immune responses (30). CD71+erythroid cells (CECs), which are immature red blood cells, including erythroblasts and reticulocytes, exert immunosuppressive functions by producing reactive oxygen species to decrease T-cell proliferation or secreting cytokines, including transforming growth factor β (TGF-β), which promotes T-cell differentiation into regulatory T cells (30, 31). In patients with cancer, anemia leads to increased frequency of CECs in the peripheral blood contributing to diminished immunity (30), and late-stage tumors can induce anemia and immunosuppressive extramedullary erythroid progenitor cells (32). In addition to affecting erythroid cells, as a systemic disease, cancer induces many functional and compositional changes to the immune system as a whole (33). Reduced abundance and decreased function of T cells in the blood was observed in cancer (33). All these studies can partly explain our results that pre-RT hemoglobin and lymphocytes were protective factors for G4 lymphopenia occurrence.

We observed that body volume was included in our final model as a protective factor for the occurrence of lymphopenia (OR, 0.56; 95% CI, 0.35–0.89, p = 0.01). In our study, body volume was defined as the part of body within the range of simulation CT scan for EBRT. On the condition of same irradiation dose, patients with larger body volume would receive lower dose per unit of body volume. We further did linear regression analysis and demonstrated that body volume was moderately correlated with BMI (R^2^ = 0.602, p <0.01). Univariate analysis of this study also showed that BMI had a propensity to protect patients from developing G4 ALC nadir (OR = 0.9, p = 0.06), which was consistent with previous studies (9, 27). Due to the significant correlation, BMI was not included in the final prediction model by the machine learning framework, while it might improve the model’s generalizability in external dataset.

Bone marrow displays structural and functional features resembling a secondary lymphoid organ and contains follicle-like structures similar to lymph nodes or spleen. Approximately 8%–20% of bone marrow mononuclear cells are lymphocytes (34). The correlation of bone marrow irradiation with lymphopenia is controversial. Some studies showed that the doses to the pelvic bone marrow was correlated with RT-induced lymphopenia (8, 35). However, a study on dosimetric predictors of lymphopenia induced by palliative RT showed that bone marrow dose–volume parameters did not predict lymphopenia. Our study did not find any relationship between DVH parameters of bones and occurrence of G4 ALC nadir. We postulate two possible reasons for the negative results of this study. First, lymphocytes are extremely radiosensitive to radiation (5), the doses to the pelvic bone exceeded the lethal dose of the lymphocyte (mean dose of bone was as high as 29.2 Gy). Second, the whole irradiated bones were treated as a whole organ during the process of our analysis. However, radiation to different parts of the pelvic bones may contribute differently to hematological toxicities. A study in patients treated with whole-pelvis RT for prostate cancer showed that the model for acute G3 lymphopenia included V40 of the whole pelvis, and the 1-year G2 lymphopenia model included V40 of the ilium (8).

It is also meaningful to explore the relationship between tumor biology and the risk of developing G4 ALC nadir. Squamous cell carcinoma accounted for approximately 80% of all cervical cancers, and adenocarcinoma accounted for approximately 20% (36). In our study, the majority of patients (95.4%) had squamous cell carcinoma, and histology types were not associated with the incidence of G4 ALC nadir (squamous vs. non-squamous: 33.1% vs. 33.3%, p > 0.999). Cho et al. also reported that histology types did not correlate with chemoradiation-induced lymphopenia in cervical cancer (p = 0.713) (4). Most cervical cancers are positive for human papillomavirus (HPV) (37). In our study, it was not possible to analyze the relationship between HPV and occurrence of G4 ALC nadir because there were a lot of missing data on HPV and we are not aware of any such analysis by others as well. With regard to FIGO stage, it had no significant impact on the occurrence of G4 ALC nadir in univariate analysis in our study, which was consistent with the results from Cho et al. (4). In FIGO (2018), locoregional lymph nodes (pelvic and paraaortic) are indicators for staging, and patients with locoregional lymph nodes metastases only are staged as IIIC. In clinical practice, radiation dose boosts mainly to locoregional metastatic lymph nodes, which was included in the final model.

With regard to ways of reducing the risk of lymphopenia or restoration of the number of lymphocytes in the peripheral blood, several measures can be attempted. First, according to our proposed model, if patients are at high risk of G4 lymphopenia, measures can be taken before treatment, such as correcting anemia, modifying EBRT plan to reduce PTV_5500 Dmax, or restoring a physiological number of lymphocytes in the peripheral blood by use of cytokines IL-2, IL-7, and IL-15, which play a role in the development, proliferation, and survival of T cells (18).

This study had some limitations. First, there were some discordances on the time points of blood tests due to the retrospective nature of the study. Second, lymphocyte subtypes changed differently after pelvic RT (38, 39) and had different impacts on treatment outcomes (40, 41). However, lymphocyte subtypes were unavailable for the patients included in the study, as lymphocyte subtypes were not routinely tested in our clinical practice, and no blood was collected for further tests. Third, body volume in the final model is determined by the extent of the simulation CT scans, which might be hard to synchronize across different centers. However, body volume was selected as a protective factor for the occurrence of G4 ALC nadir through elastic-net regression modeling; we think it is still meaningful to keep it in the final model to remind readers of its potential role in the occurrence of G4 ALC nadir during CCRT in cervical cancer, and we also recommend external validation of the role of body volume. Fourth, although the data were split into a training and a testing set, it would be better to use external data from different institutions to validate our results.

In conclusion, the present study developed and validated a comprehensive model integrating clinical and dosimetric parameters by machine learning method, which performed well in predicting G4 lymphopenia during pelvic RT for cervical cancer and may facilitate physicians to identify patients at high risk of G4 lymphopenia who might benefit from modified treatment approaches.

## Data availability statement

The raw data supporting the conclusions of this article will be made available by the authors, without undue reservation.

## Ethics statement

This study was reviewed and approved by Ethics committee of the University of Hong Kong-Shenzhen Hospital. The ethics committee waived the requirement of written informed consent for participation.

## Author contributions

LY: Primary data collection, data analysis, manuscript writing, and manuscript approval. ZX: Primary data collection, data analysis, manuscript editing, and manuscript approval. HY: Corresponding author, data double check, data analysis, result interpretation, study design, and manuscript approval. LG: Corresponding author, study design oversight, data quality control, results check and manuscript approval. All authors contributed to the article and approved the submitted version.

## Funding

This project is supported in part by Health Commission of Guangdong Province, China (NO. B2020100); Shenzhen Science and Technology Program (NO. KQTD20180411185028798 and JCYJ20210324114600002); High Level-Hospital Program, Health Commission of Guangdong Province, China (NO. HKUSZH201902031 and HKUSZH201901038); Shenzhen Fundamental Research Program (NO. JCYJ2020109150427184) and Shenzhen Key Medical Discipline Construction Fund (NO. SZXK014).

## Conflict of interest

The authors declare that the research was conducted in the absence of any commercial or financial relationships that could be construed as a potential conflict of interest.

## Publisher’s note

All claims expressed in this article are solely those of the authors and do not necessarily represent those of their affiliated organizations, or those of the publisher, the editors and the reviewers. Any product that may be evaluated in this article, or claim that may be made by its manufacturer, is not guaranteed or endorsed by the publisher.

## Supplementary material

Supplementary Figure 1 The Pearson’s correlations among clinical characteristics and dose-volume histogram (DVH) metrics.

Supplementary Figure 2 The selected frequencies of 14 clinical characteristics and 63 dose-volume histogram (DVH) parameters in Elastic-net regression models in 100 iterations bootstrapping.
