# Supplementary figures and images for: A machine learning model for grade 4 lymphopenia prediction during pelvic radiotherapy in patients with cervical cancer
Source: Front Oncol. 2022 Sep 15;12:905222. doi: 10.3389/fonc.2022.905222 (PMC9524190; doi:10.3389/fonc.2022.905222)

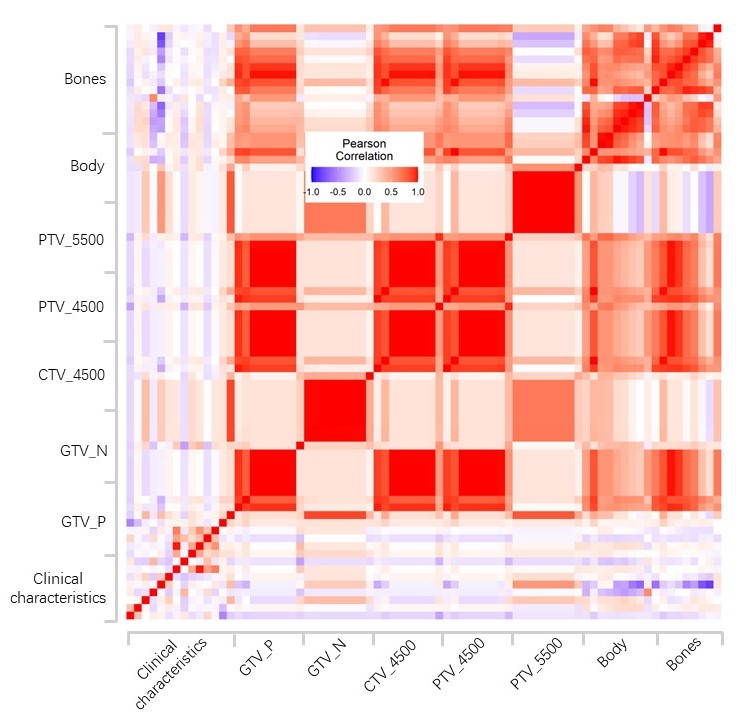

Supplement: Supplementary Figure 1 — The Pearson’s correlations among clinical characteristics and dose-volume histogram (DVH) metrics. [file Image_1.jpeg]

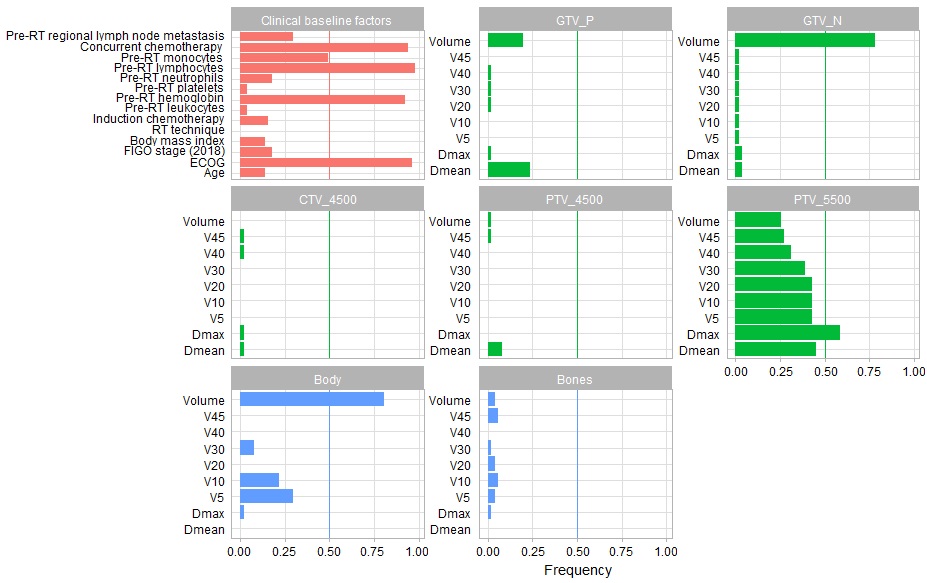

Supplement: Supplementary Figure 2 — The selected frequencies of 14 clinical characteristics and 63 dose-volume histogram (DVH) parameters in Elastic-net regression models in 100 iterations bootstrapping. [file Image_2.jpeg]
