# Supplementary material for: A machine learning model for grade 4 lymphopenia prediction during pelvic radiotherapy in patients with cervical cancer
Source: Front Oncol. 2022 Sep 15;12:905222. doi: 10.3389/fonc.2022.905222 (PMC9524190; doi:10.3389/fonc.2022.905222)
Supplement: Supplementary file 3 [file Table_1.docx]

|  | Supplemental table 1. Univariate analysis of clinical features for G4 ALC nadir | | | | | |
| --- | --- | --- | --- | --- | --- | --- |
| Features | | Categories | OR | 95%CI | P-value | Adjusted P-value* |
| Age (years) | | Continuous | 0.97 | 0.93-1 | 0.03 | 0.45 |
| ECOG | | 0-1 | reference |  |  |  |
|  |  | 2 | 0.11 | 6.53-2e-03 | 0.04 | 0.6 |
| FIGO stage | | I-II | reference |  |  |  |
|  |  | III | 2.15 | 0.08-56.8 | 0.1 | 1 |
|  |  | IV | 1.5 | 0.55-4.06 | 0.61 | 1 |
| Body mass index | | Continuous | 0.9 | 0.8-1.01 | 0.06 | 0.9 |
| RT technique | | 3D-CRT | reference |  |  |  |
|  |  | RapidArc | 1.54 | 0.27-8.67 | 0.38 | 1 |
| Induction chemotherapy | | Without | reference |  |  |  |
|  |  | With | 1.09 | 0.76-1.57 | 0.85 | 1 |
| Concurrent chemotherapy | | Without | reference |  |  |  |
|  |  | With | 5.73 | 0.07-489.33 | 0.02 | 0.3 |
| Pre-RT regional LN metastasis | | Without | reference |  |  |  |
|  |  | With | 2.22 | 0.05-90.4 | 0.06 | 0.9 |
| Pre-RT leukocytes | | Continuous | 0.98 | 0.89-1.08 | 0.72 | 1 |
| Pre-RT hemoglobin | | Continuous | 0.97 | 0.95-0.99 | 5.6e-03 | 0.08 |
| Pre-RT platelets | | Continuous | 1 | 1-1 | 0.71 | 1 |
| Pre-RT neutrophils | | Continuous | 1.01 | 0.91-1.13 | 0.82 | 1 |
| Pre-RT lymphocytes | | Continuous | 0.22 | 0.1-0.49 | 2.0e-04 | 0.003 |
| Pre-RT monocytes | | Continuous | 1.32 | 0.32-5.47 | 0.71 | 1 |
| CI, confidence interval; ECOG, Eastern Cooperative Oncology Group; FIGO, International Federation of Gynecology and Obstetrics; LN, lymph node; OR, odds ratio; RT, radiotherapy; 3D-CRT, three-dimensional conformal radiotherapy.  * The P-value was adjusted by Bonferroni corrected. | | | | | | |

| Supplemental table 2. Summary of DVH metrics | | | | | | | |
| --- | --- | --- | --- | --- | --- | --- | --- |
| Features | DVH metrics of both tumor targets and organs at risk, Median (IQR) | | | | | | |
|  | GTV_P | GTV_N | CTV_4500 | PTV_4500 | PTV_5500 | Body | Bones |
| Dmean (Gy) | 47.1 (46.6-49.2) | 56.5 (0-57.3) | 47.9 (47-50.8) | 47.6 (46.8-50) | 56.1 (0-56.8) | 12 (10.5-13.9) | 29.2 (27.7-31.8) |
| Dmax (Gy) | 52.7 (48.5-57.8) | 57.4 (0-58.5) | 58.4 (54.6-59.2) | 58.5 (54.9-59.2) | 57.7 (0-58.9) | 58.5 (54.9-59.2) | 58.1 (53.6-58.8) |
| V5 (%) | 100 (100-100) | 100 (0-100) | 100 (100-100) | 100 (100-100) | 100 (0-100) | 45.3 (42.1-49.4) | 96.8 (95.6-98.5) |
| V10 (%) | 100 (100-100) | 100 (0-100) | 100 (100-100) | 100 (100-100) | 100 (0-100) | 38.4 (35.4-42.6) | 89.2 (86.3-92.7) |
| V20 (%) | 100 (100-100) | 100 (0-100) | 100 (100-100) | 100 (100-100) | 100 (0-100) | 25.4 (21.4-30.8) | 72.7 (69.2-77.6) |
| V30 (%) | 100 (100-100) | 100 (0-100) | 100 (100-100) | 100 (100-100) | 100 (0-100) | 14.9 (11.9-18.3) | 51.7 (47.1-56.8) |
| V40 (%) | 100 (100-100) | 100 (0-100) | 100 (100-100) | 100 (100-100) | 100 (0-100) | 9.1 (7.3-12.5) | 27.5 (23.2-35.1) |
| V45 (%) | 100 (99.9-100) | 100 (0-100) | 100 (99.9-100) | 99.3 (98.3-99.8) | 100 (0-100) | 6.7 (5.3-9.4) | 13.5 (10.5-20) |
| Volume (cc) | 73.4  (49.4-122.1) | 1.4  (0-5.2) | 888.9  (773.8-1.03e+03) | 1.37e+03  (1.24e+03-1.54e+03) | 18.2  (0-58.2) | 2.44e+04  (2.09e+04-2.83e+04) | 1.17e+03  (1.07e+03-1.28e+03) |
| cc, cubic centimeter; CTV_4500, clinical target volume receiving prescription dose of ≥45Gy; Dmax, maximum dose; Dmean, mean dose; DVH, dose-volume histogram; GTV_P, gross tumor volume of primary tumor; GTV_N, gross tumor volume of regional metastatic lymph nodes; IQR, interquartile range; PTV_4500, planning target volume receiving prescription dose of ≥45Gy; PTV_5500, planning target volume receiving prescription dose of ≥55Gy; V5, V10, V20, V30, V40, and V45, the percentage of the whole volume receiving ≥ 5Gy, ≥ 10Gy, ≥ 20Gy, ≥ 30Gy, ≥ 40Gy, and ≥ 45Gy, respectively. | | | | | | | |

| Supplemental table 3. Univariate analysis of DVH metrics for G4 ALC nadir | | | | | | | | | | | | | | | | | | | | | | | | | | | | |
| --- | --- | --- | --- | --- | --- | --- | --- | --- | --- | --- | --- | --- | --- | --- | --- | --- | --- | --- | --- | --- | --- | --- | --- | --- | --- | --- | --- | --- |
| Features | GTV_P | | | | GTV_N | | | | CTV_4500 | | | | PTV_4500 | | | | PTV_5500 | | | | Body | | | | Bones | | | |
|  | OR | 95%CI | P | Ad. P | OR | 95%CI | P | Ad. P | OR | 95%CI | P | Ad. P | OR | 95%CI | P | Ad. P | OR | 95%CI | P | Ad. P | OR | 95%CI | P | Ad. P | OR | 95%CI | P | Ad. P |
| Dmean (Gy) | 0.98 | 0.93-1.04 | 0.48 | 1 | 1.01 | 0.99-1.02 | 0.38 | 1 | 0.98 | 0.94-1.04 | 0.56 | 1 | 0.98 | 0.93-1.04 | 0.52 | 1 | 1.01 | 1-1.03 | **0.05** | 1 | 1.08 | 0.96-1.22 | 0.19 | 1 | 0.99 | 0.92-1.05 | 0.65 | 1 |
| Dmax (Gy) | 1.01 | 0.96-1.05 | 0.77 | 1 | 1.01 | 0.99-1.02 | 0.38 | 1 | 1.01 | 0.96-1.05 | 0.76 | 1 | 1.01 | 0.96-1.05 | 0.77 | 1 | 1.01 | 1-1.03 | **0.05** | 1 | 1.01 | 0.96-1.05 | 0.79 | 1 | 1.01 | 0.96-1.06 | 0.72 | 1 |
| V5 (%) | 0.99 | 0.97-1.02 | 0.62 | 1 | 1 | 1-1.01 | 0.34 | 1 | 0.99 | 0.97-1.02 | 0.62 | 1 | 0.99 | 0.97-1.02 | 0.62 | 1 | 1.01 | 1-1.02 | **0.05** | 1 | 1.03 | 0.99-1.07 | **0.09** | 1 | 0.99 | 0.96-1.02 | 0.56 | 1 |
| V10 (%) | 0.99 | 0.97-1.02 | 0.62 | 1 | 1 | 1-1.01 | 0.34 | 1 | 0.99 | 0.97-1.02 | 0.62 | 1 | 0.99 | 0.97-1.02 | 0.62 | 1 | 1.01 | 1-1.02 | **0.05** | 1 | 1.04 | 0.99-1.09 | **0.09** | 1 | 0.99 | 0.96-1.02 | 0.56 | 1 |
| V20 (%) | 0.99 | 0.97-1.02 | 0.62 | 1 | 1 | 1-1.01 | 0.34 | 1 | 0.99 | 0.97-1.02 | 0.62 | 1 | 0.99 | 0.97-1.02 | 0.61 | 1 | 1.01 | 1-1.02 | **0.05** | 1 | 1.03 | 0.98-1.09 | 0.23 | 1 | 0.99 | 0.96-1.02 | 0.51 | 1 |
| V30 (%) | 0.99 | 0.97-1.02 | 0.62 | 1 | 1 | 1-1.01 | 0.34 | 1 | 0.99 | 0.97-1.02 | 0.62 | 1 | 0.99 | 0.97-1.02 | 0.61 | 1 | 1.01 | 1-1.02 | **0.05** | 1 | 1.05 | 0.98-1.13 | 0.16 | 1 | 1 | 0.97-1.04 | 0.88 | 1 |
| V40 (%) | 0.99 | 0.97-1.02 | 0.62 | 1 | 1 | 1-1.01 | 0.34 | 1 | 0.99 | 0.97-1.02 | 0.62 | 1 | 0.99 | 0.97-1.02 | 0.61 | 1 | 1.01 | 1-1.02 | **0.05** | 1 | 1.03 | 0.95-1.13 | 0.46 | 1 | 1 | 0.97-1.03 | 0.92 | 1 |
| V45 (%) | 0.99 | 0.97-1.02 | 0.65 | 1 | 1 | 1-1.01 | 0.34 | 1 | 0.99 | 0.97-1.02 | 0.62 | 1 | 0.99 | 0.96-1.02 | 0.59 | 1 | 1.01 | 1-1.02 | **0.05** | 1 | 1.01 | 0.92-1.12 | 0.79 | 1 | 0.99 | 0.96-1.02 | 0.55 | 1 |
| Volume (cc) | 1 | 1-1.01 | **0.05** | 1 | 1.07 | 1.01-1.12 | **0.01** | 0.63 | 1 | 1-1 | 0.16 | 1 | 1 | 1-1 | 0.27 | 1 | 1 | 1-1.01 | **0.05** | 1 | 1 | 1-1 | **0.06** | 1 | 1 | 1-1 | 0.92 | 1 |
| Ad. P, adjusted P value by Bonferroni corrections; cc, cubic centimeter; CI, confidence interval; CTV_4500, clinical target volume receiving prescription dose of ≥45Gy; Dmax, maximum dose; Dmean, mean dose; GTV_P, gross tumor volume of primary tumor; GTV_N, gross tumor volume of locoregional metastatic lymph nodes; OR, odds ratio; PTV_4500, planning target volume receiving prescription dose of ≥45Gy; PTV_5500, planning target volume receiving prescription dose of ≥55Gy; V5, V10, V20, V30, V40, and V45, the percentage of the whole volume receiving ≥ 5Gy, ≥ 10Gy, ≥ 20Gy, ≥ 30Gy, ≥ 40Gy, and ≥ 45Gy, respectively. | | | | | | | | | | | | | | | | | | | | | | | | | | | | |
